# Milled Stress Reduces Morphine-Induced Locomotion in F2 NMRI Mice

**Published:** 2011

**Authors:** Hassan Ghoshooni, Pooya Payandeh Mehr, Seyyed Hosein Salimi, Leila Golmanesh, Ahamadreza Dehpour, Hedayat Sahraei

**Affiliations:** a *Neuro science Research Center, Baqyiatallah(A.S).University of Medical Sciences, Tehran, Iran*; b *Department of Pharmacology, Faculty of Medicine, Tehran University of Medical Sciences, Tehran, Iran.*; c *Department of Psychology, School of Medicine, Baqyiatallah(A.S). University of Medical Sciences, Tehran, Iran. *; d *Molecular Biology Research Center, Baqyiatallah(A.S). University of Medical Sciences, Tehran, Iran.*

**Keywords:** Stress, Motor Activity, F_2_ generation, Morphine, Female mice

## Abstract

In the present study, the effects of pregnant NMRI mice restraint stress on the responsibility of their children to the behavioral properties of morphine, sulpiride and dextromethorphan were investigated in the F_2_ generation.

Twenty four pregnant NMRI female mice (W: 25 g) were divided into the experimental and control groups (n = 12/group). Animals in the experimental group were kept in the restraint cylinder (ID = 6 cm, L = 20 cm) for 60 min/day for 15 consecutive days, while the control group did not receive stress. On the 8^th^ day, blood samples were taken from the retro-orbital of both groups for corticosterone measurement (ELYSA method). After the F_2_ weight gained 20-25 g, their tendency for right-handedness or Left-handedness and response to the new environment was determined by T-maze and open field method, respectively. In addition, the effects of morphine, sulpiride and dextromethorphan on the animals’ motor activity were studied.

Results showed that plasma corticosterone level in the experimental group was elevated significantly with respect to the controls. In the off-springs, left-handedness was more frequent in both the male and female animals whose mothers experienced restrained stress. In the open field paradigm, however, the females of experimental group showed more activity compared to the others. While the females of the control group showed more response to morphine (50 mg/Kg), interestingly, both male and female animals in the experimental group showed hypo activity to morphine (0.5, 5, and 50 mg/Kg). Similarly, sulpiride (25 and 50 mg/Kg) reduced the animals’ activity in both groups, while dextromethorphan did not cause any difference. In conclusion, it can be summarized that stress during the gestation period may change the response to the morphine-induced motor activity, in a sex-dependent manner.

## Introduction

Stress is considered as an important factor which can influence embryos growth during the gestation period of life. Stress has been defined as any condition that changes internal or external melio. Stress reaction differs in accordance with its severity to threats in life (1, 2). In general, it is accepted that stress can induce a neuroendocrine response originated from hypothalamus and ends in adrenal gland (3). Corticosterone and norepinephrine released from rodents’ adrenal cortex and medulla respectively, prepare the animals for ameliorate the menace and/or overcome it (3). However, when a pregnant animal is exposed to a stressful event, with a possible increase in the plasma concentration of its corticosterone, the overloaded hormone may readily cross the placenta barrier and affect its embryos growth and development (4, 5). In this regard, studies in rodents indicated that the prenatal stress increased the anxiety-related behaviors (6). In addition, prenatal stress may be linked to a typical laterality in rats (7-9). Investigators also revealed that the early life stress can alter dopaminergic system activity in rats (4), which may influence their response to the psycho stimulants, indicating the importance of early life events on later brain activities. All together, these findings indicated that implication of stress to a pregnant animal could lead to a serious abnormal brain function of its off-springs. However, despite the intense investigations, regarding to the effects of prenatal stress on the function and morphology of the brain dopaminergic system (4, 9, 10) and the effects of postnatal stress on the dopaminergic (9) and opioidergic system (11, 12), there is no study indicating the role of a maternal mild stress on the response of the off-springs to morphine-induced locomotion in mice. Such study may provide more information to reach a better understanding of what may occur in brain’s opioidergic system after the prenatal stress.

## Experimental

*Animals*

Thirty-two male and female NMRI mice (Pasture Institute, Tehran, Iran) were housed one by one in a mating cage (15*×*15*×*20 cm) over the night for mating. At 8:0.0 PM the following day, the animals were separated and the Embryonic Zero day (E0) was detected through observing the vaginal plug and/or identifying the sperm in vaginal smear. The pregnant mice were randomly divided into control and experimental groups (n = 12/group). The animals were housed 2/cage and remained in a standard animal room conditions (22 ± 2^°^C and 50% humidity with 12 h light/dark cycle) and have free access to standard mouse chow and tap water add lib except during the experiments.

*Experimental procedure: Stress protocol in pregnant mice (i.e. F*_1_ *generation)*

All experiments took place in light time (10:00-12:00). To induct the stress, each animal was placed in a PVC cylinder (20 cm length, 6.5 cm diameter) in which one of the ends was blocked (13, 14) and the other one was sealed by pieces of plastic. In such apparatus, animals could not move easily though their abdomens were not under the pressure. The pregnant mice were placed in these vacuums 1 h/day for the next 15 days (Embryonic day 15) (13, 14). The time of stress exposure was randomly designed for minimum stress adaptations. On E16, the stress was interrupted and animals were kept for delivering their pups and nursing them.

*Blood sampling and plasma corticosterone levels detection in stressed pregnant mice*

Blood samples were taken from retro-orbital sinus (0.1 mL of the blood in 0.9 mL sodium citrate 5%) of the pregnant mice on Embryonic day 8 (E8) of pregnancy between 10:00-12:00 (13, 14). The samples were centrifuged in 3000 rpm for 5 min in 4°C and the supper natant serum was collected for corticosterone detection. Corticosterone concentration was determined by ELISA kit (Rat Corticosterone ELISA kit; EIA-4164; DRG Instruments GmbH, Germany) in 450 nm.

Determination of left-handedness and right-handedness in F2 generation When the off-springs’ (both males and females) weight reached up to 20 g, the animals were placed in a T-maze apparatus and their tendency for left or right arm of the maze was estimated (n = 30 for each session/sex; 4 groups with total number of 120 mice). All experiments were taken place in a silent room from 10:0.0 AM to 17:0.0 PM. The experiment was repeated three times for each animal and the animal’s arm which was selected more than once (i.e., 2 of 3), was considered as its tendency. The experiments took place in separate rooms for males and females.

*Locomotion activity in F*_2_ *generation*

After right-handedness and left-handedness determination, the next generations (*e.g.* F_2_) were engaged in locomotor activity experiments. All experiments took place in a silent room from 10:0.0 AM till 17:0.0 PM (15). Male and female F_2_ generation of the experiment or control mothers (n = 7-8/group, total number = 131 for males and 140 for females) were brought to the lab and remain there 1 h for adaptation. These animals were different from the animals used in the left-handedness and right-handedness determination. Each animal was placed in an open field apparatus (a cylinder with 30 cm diameter and 30 cm high made by Plexiglas), and after 5 min, its locomotion activity was recorded for 10 min by a digital camera. These experiments also took place in separate rooms for male and female mice.

*Drugs*

Morphine sulfate (TEMAD Co., Tehran, Iran), ± sulpiride ([±]-N-1-[Ethylpyrrolidin-2-ylmethyl])-2-methoxy-5-sulfamoylbenzamide), (Sigma Chemical Co., California, USA) and dextromethorphan hydrobromide (TOCRIS, UK) were dissolved in normal saline (10 mL) and were injected in volume of 0.1 mL/10 g per body weight. Morphine was injected subcutaneously (s.c.). Sulpiride and dextromethorphan were injected intra-peritoneally (IP) 30 min before the beginning of experiments. The doses for all drugs were chosen according to the previous studies (15-17).

*Statistical analysis*

Data are shown as mean ± SEM. One-way analysis of variance (ANOVA) followed by Tukey post-test, Un-Paired t-test or chi-square tests was applied. p < 0.05 considered statistically significant.

## Results and Discussion

*Effects of stress on plasma corticosterone level in pregnant mice*

**Figure 1:**
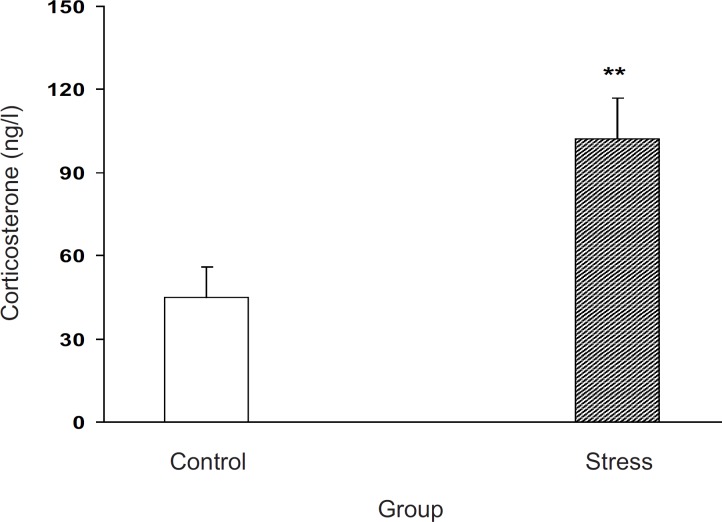
Plasma corticosterone level increment after milled restraint stress in female pregnant mice on E8 Plasma corticosterone level was increased in the experimental group. Data showed as mean ± SEM, **: p < 0.01, which proves different from control group

The effect of stress on blood corticosterone level in pregnant animals is shown in Figure 1. Our results indicated that milled stress can be efficient for the elevation of corticosterone level in the experimental group for about 300%, which was statistically significant (p < 0.01, Figure 1).

*Determination of right or left* *-* *handedness in off* *-* *springs*

**Figure 2:**
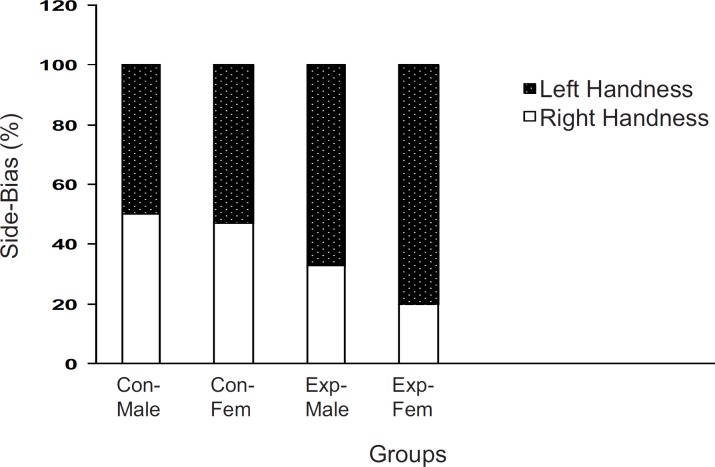
Brain laterality in F2 generation of stressed and nonstressed pregnant mice The animals were examined in a T-maze equipped for determination of left- handedness or right-handedness. The animals of experimental group showed more side biased compared to the controls and it was more significant in the females. Animals data are showed as % in each group (Con = Control; Exp = Experimental; Fem = Female).

When the off-springs of the control group were placed in T-maze, more than 80% of the males and females preferred the right arm . However, there sults of off-springs in experimental mothers were completely reversed as up to 70% of both males and females preferred their left arm to the right one (p < 0.001, Figure 2). 

*Exploratory behavior in non-treated F*_2_ *generation mice*

**Figure 3:**
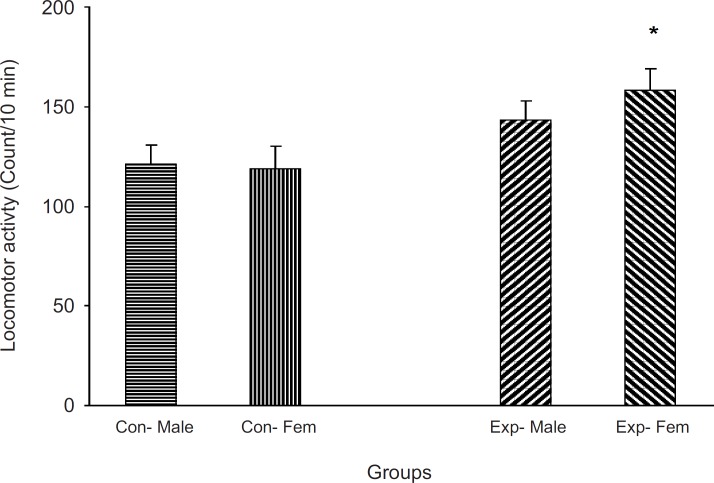
The F2 generation behavior in non-familiar environment The animals did not receive any treatment and were allowed to freely move in a cylinder for 5 min and then their activity was measured

Animals which did not receive a treatment were located in an open field apparatus and their activity was measured for 10 min. Results showed that the experimental groups were more active than the control one (p < 0.05, Figure 3)

for additional 10 min. The animals of experimental group showed more activity than the normal situation. Data showed as mean ± SEM,*: p < 0.05 proved different from controls (Con = Control; Exp = Experimental; Fem = Female).

*Morphine induced locomotion in off-springs*

**Figure 4:**
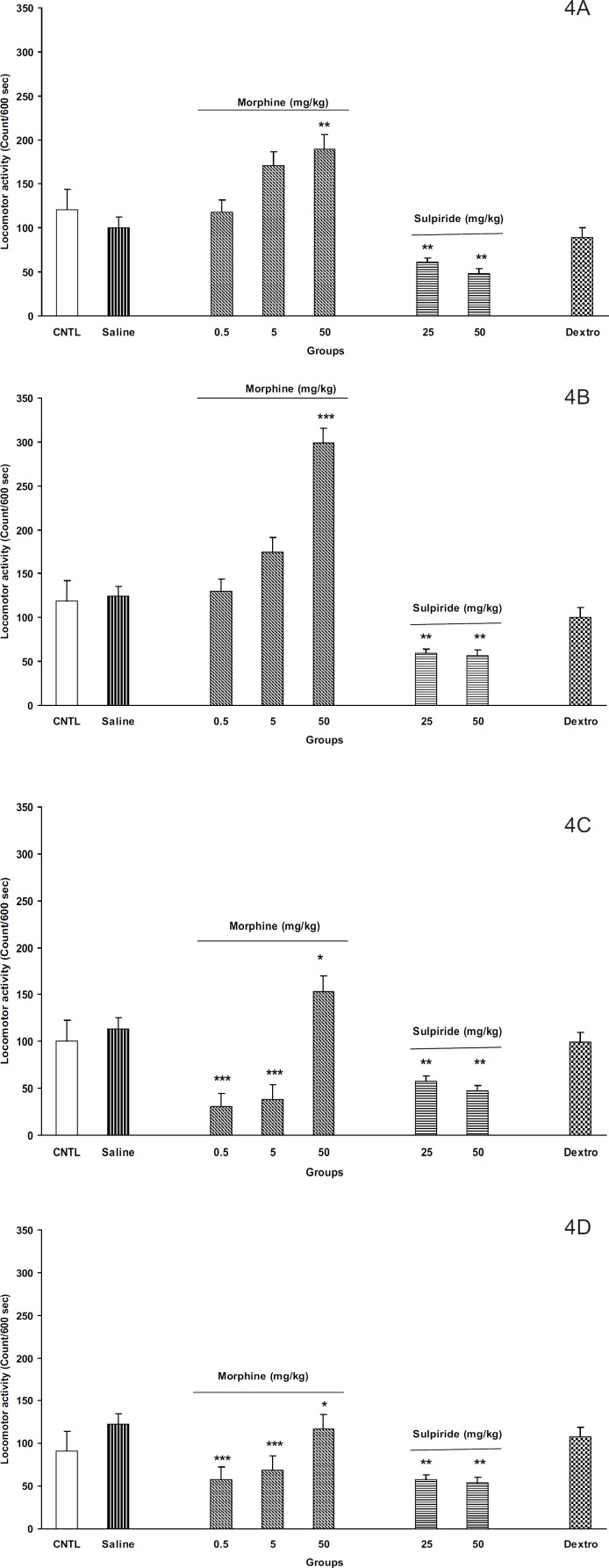
The F2 generation response to morphine , sulpiride and dextromethorphan in the control and the experimental groups. A: male, control; B: male, experimental; C: female, control; and D: female experimental. Animals received different doses of morphine (0.5, 5 and 50 mg/Kg), sulpiride (25 and 50 mg/Kg) or dextromethorphan (20 mg/Kg) and were placed in open filed apparatus and after and 5 min their activity was recorded for 10 min. Data showed as mean ± SEM; *: p < 0.05; **: p < 0.01; ***: p < 0.001 proved different from controls . (CNTL= control, without any injection; Dextro=Dextromethorphan

As shown in Figures 4A-4D, the administration of different doses of morphine (0.5, 5, and 50 mg/Kg) induced locomotor activity in the off-springs of control mothers (Figure 4A for males and Figure 4B for females). Moreover, maximum response was achieved in dose of 50 mg/Kg of morphine in both animals. In addition, it seems that females were more responsible to morphine than males (Figures 4A and 4B, p < 0.001).

On the other hand, our results for the off-springs of experimental mothers revealed that morphine induces hypoactivity in doses 0.5 and 5 mg/Kg in both sexes (Figures 4C and 4D, p < 0.0001). Interestingly, the females of the experimental group also showed hypo activity for dose of 50 mg/Kg of morphine instead of hyper activity induced by the same dose in males (Figures 4C and 4D).

By injecting different doses of dopamine D_2_ receptor antagonist, sulpiride (25 and 50 mg/Kg, SC) induced hypoactivity in all animals (Figures 4A, 4B, 4C, and 4D, p < 0.001). However, the *N- *methyl-*D-* aspartate (NMDA) glutamate receptor antagonist, dextromethorphan, (20 mg/Kg, IP) did not change the animals’ locomotor activity in all groups.

First of all, we found that psychological stress (*i.e*. restraint in a closed tube) can increase plasma corticosterone level in the pregnant mice. Second, the study revealed that off-springs from stressed mothers had different tendency for left or right preference, and third, off-spring’s responsibility to morphine was different according to their sex.

Our results showed that corticosterone plasma level was elevated in the experimental group which indicated the method effectiveness for stress induction. It is now accepted that incrementing in plasma corticosterone level is an indicator of stress response in rodents (3, 18, 19), which further indicates the involvement of HPA axis activity as the main stress response element (2, 20). In agreement with our results, it is clear that immobilization of male rats also increases plasma corticosterone level (13, 14, 21). Our results further indicated that the milled restraint stress in the female pregnant mice canincrease the plasma corticosterone level as well.

Previous studies have indicated that intra-uterine stress can affect the brain laterality (22-24) and may be linked to drug abuse (11, 12, 25). Our results showed that left-handedness and right-handedness was equal in both males and females in the control group whereas it was a shift to left-handedness in both males and females in the experimental group. It seems that female mice are more sensitive to intra-uterine stress, in comparison with the males. However, the exact reason for the observed results is not available and needs further experiments. These results further indicated that a milled restraint stress which can increase plasma corticosterone level in the pregnant mice, can affect the brain laterality in the embryos in the way that the F_2_ generation showed the complete diversity from the controls. In a similar line with previous studies, it can be concluded that some regions within the brain of the experimental F_2_ generation are not well developed (4, 9, 24, 26-28). Moreover, as it was mentioned earlier, it may also be related to drug abuse. In our experiment, the animal’s response to the new environment was also different in experimental and control groups. Knowing the response of the animals to the new environment is the aim of several studies. For example, it is clear that animals show more activity in the new environment (10), and this is the result of dopamine mesolimbic activity (10). Based on available data, it is important that animals with prenatal stress experiences may suffer from abnormal dopamine mesolimbic system development (4, 9, 28-32). It is likely that corticosterone plasma level increment resulted from restraint stress in the experimental group can interact with factors which influence fetal brain development and the change of locomotion in the new environment is the result.

The response of F_2_ generation to morphine (as a typical opioid) also showed difference in the experimental group. While the controls showed no response to low doses of morphine, the response of the experimental group was even a decline. In agreement with our results, Michaels *et al.* showed that the early post-natal stress in rats can change the response to the mu-opioid receptor agonists in place conditioning paradigm (12). In addition, Moffett has shown that post-natal stress can also alter the cocaine self-administration in the rats (33). Alteration in response to morphine may be linked to dopamine mesolimbic system abnormal development and/or opioid receptor abnormality. It must be mentioned that the morphine’s ability of increasing the locomotion activity is hypothesized to be built base on dopamine mesolimbic activity (34-38). However, as mentioned above, experiments indicated that pre-natal and post-natal stress can impair the development of mesolimbic dopamine system (4, 9). Based on these findings, one may conclude that our experiments, in which the prenatal stress increases the maternal plasma corticosterone level, leads to defect in brain mesolimbic dopamine system development and the response to morphine differs from controls as a result. On the other hand, some contradict data indicated that the response to the opioid agonists was changed in rats experienced early post-natal stress (12), which also may be true for the response observed in the present study.

In completion, our results obtained from D_2_ dopamine antagonist, sulpiride, may be useful for resolving the above theories. Our results indicated that sulpiride reduced the locomotion activity in all groups. The dopamine D_2_ receptors have shown to be involved in locomotion and their inhibition leads to hypo activity in both human and animal models (15, 39, 40, 41). Our results are in agreement with these observations and indicate that dopamine D_2_ receptors are functional in all groups. Considering the results, it concluded that in experimental group, the mesolimbic dopamine system responsiveness to morphine may be different from that of dopamine D_2_ receptors.

In the last part of our experiments, dextromethorphan as *N-* methyl-*D- *aspartate (NMDA) receptor antagonist (17) was administered to the animals for investigation of possible brain glutamate system involvement and/or alteration in response to intra-uterine stress. However, no response was observed when the drug was administrated. Our results did not regret the role of brain glutamate system in the alteration in response to opioids, even thought other methods such as place conditioning paradigm and self-administration procedure may be useful for further evaluation of the role of this system.

In conclusion, results indicated that first, F_2_ generation brain laterality was changed in animals whose mothers experienced milled stress. Second, response to morphine-induced locomotion was also altered in the experimental group which was sex dependent but could not be linked to D_2_ dopamine and NMDA glutamate receptor dysfunction.
